# Network analysis of human protein location

**DOI:** 10.1186/1471-2105-11-S7-S9

**Published:** 2010-10-15

**Authors:** Gaurav Kumar, Shoba Ranganathan

**Affiliations:** 1 ARC Centre of Excellence in Bioinformatics and Department of Chemistry and Biomolecular Sciences, Macquarie University, Sydney NSW, Australia; 2 Department of Biochemistry, Yong Loo Lin School of Medicine, National University of Singapore, Singapore

## Abstract

**Background:**

Understanding cellular systems requires the knowledge of a protein's subcellular localization (SCL). Although experimental and predicted data for protein SCL are archived in various databases, SCL prediction remains a non-trivial problem in genome annotation. Current SCL prediction tools use amino-acid sequence features and text mining approaches. A comprehensive analysis of protein SCL in human PPI and metabolic networks for various subcellular compartments is necessary for developing a robust SCL prediction methodology.

**Results:**

Based on protein-protein interaction (PPI) and metabolite-linked protein interaction (MLPI) networks of proteins, we have compared, contrasted and analysed the statistical properties across different subcellular compartments. We integrated PPI and metabolic datasets with SCL information of human proteins from LOCATE and GOA (Gene Ontology Annotation) and estimated three statistical properties: Chi-square (χ^2^) test, Paired Localisation Correlation Profile (PLCP) and network topological measures. For the PPI network, Pearson's chi-square test shows that for the same SCL category, twice as many interacting protein pairs are observed than estimated when compared to non-interacting protein pairs (χ^2 ^= 1270.19, *P-value *< 2.2 × 10^-16^), whereas for MLPI, metabolite-linked protein pairs having the same SCL are observed 20% more than expected, compared to non-metabolite linked proteins (χ^2 ^= 110.02, *P-value *< 2.2 x10^-16^). To address the issue of proteins with multiple SCLs, we have specifically used the PLCP (Pair Localization Correlation Profile) measure. PLCP analysis revealed that protein interactions are majorly restricted to the same SCL, though significant cross-compartment interactions are seen for nuclear proteins. Metabolite-linked protein pairs are restricted to specific compartments such as the mitochondrion (*P-value *< 6.0e-07), the lysosome (*P-value *< 4.7e-05) and the Golgi apparatus (*P-value *< 1.0e-15). These findings indicate that the metabolic network adds value to the information in the PPI network for the localisation process of proteins in human subcellular compartments.

**Conclusions:**

The MLPI network differs significantly from the PPI network in its SCL distribution. The PPI network shows passive protein interaction, possibly due to its high false positive rate, across different subcellular compartments, which seem to be absent in the MLPI network, as the MLPI network has evolved to maintain high substrate specificity for proteins.

## Background

The eukaryotic cell consists of many different subcellular compartments or organelles. Most of the cellular functions critical to the cell's survival are performed by proteins inside the cell. A typical cell thus contains a large number of protein molecules that are resident in specific compartments or organelles, referred to as "subcellular locations" (SCL). The major compartments, according to the Gene Ontology Consortium, are: cell surface, chromosome, cytoplasm, cytoskeleton, cytosol, endosome, endoplasmic reticulum, extracellular region, Golgi apparatus, membrane, mitochondria, nucleus, spliceosome, ribosome, vacuoles and organelle lumen [1]. These subcellular compartments are further refined into more specific compartments.

The functions of proteins are determined by specific physico-chemical environment present inside various compartments or organelles. Therefore, it is important to identify the SCL of each protein, for understanding its functional and cellular role. While protein SCL can be determined by biochemical experimentation, with the growing number of new protein sequences in the post-genomic era, experimental characterization of SCL is available for only 11.1% of the total protein sequences present in the UniProt Knowledge Base (version 57.9) [2]. For human proteins, the number is slightly better, with 34.1% having SCL annotations (Table 1). There is thus a huge gap between protein sequences with and without SCL annotation, necessitating computational approaches to predict the SCL from sequence information.

**Table 1:** Summary of SCL annotation in UniProtKB.

Items	Description	No. of Protein Sequences	Dataset Size	%
A	Proteins with SCL annotation in UniProt database	274730	494762	55.52
B	Proteins in A with experimentally known SCL	55079	494762	11.13
C	Proteins in A with uncertain terms such as potential/probable/similarity	219651	494762	44.39
D	Proteins with GO annotation	461365	494762	93.24
E	Protein with SCL annotation in GO database	337762	494762	68.26
F	UniProt human entries with experimentally known SCL	6923	20274	34.14
G	UniProt human entries with uncertain terms such as potential/probable/similarity	7486	20274	36.92

Distribution of 494762 protein entries from UniProtKB/Swiss-Prot* database (version 57.9) according to their SCL annotation and GO database reference.

* The original number of UniProt protein entries was 510076. Of these, 15314 were annotated as "fragment" or contained less than 50 amino acids residues, hence, were removed from further consideration, i.e. 494762. Similarly, we considered only 20274 human protein entries out of 20334 sequences.

Early computational methods were restricted to specific subcellular compartments and depended on sequence information alone [3]. Protein sequence information comprises amino-acid composition, their physico-chemical properties (such as molecular weight, hydrophobicity, side-chain mass and amino-acid propensity), protein motifs, signal peptides and functional domain composition. However, given the variety of accepted subcellular locations that are functionally essential to completely characterize a protein, novel approaches such as machine learning and text mining have improved SCL predictability [3,4]. A machine-learning method relies on the recognition of patterns that are best characterized on the set of proteins whose localisation are known. A few studies use a systems biology approach for the prediction of a protein's SCL [5], adopting an integrated methodology of high-throughput proteomic data such as protein-protein interaction (PPI) networks and protein motifs to understand and predict the SCL of a eukaryotic protein [5,6].

The use of PPI network to predict function relies on the principal assumption that the interacting protein pairs are likely to collaborate for a common purpose and have to be in close proximity in order to interact. Schwikowski *et al. *[7] were the first to show that the *Saccharomyces cerevisiae *PPI network could be used to classify protein SCL based on the idea of "guilt by association or neighbouring count method". Their approach correctly identifies 76% of the interacting protein pairs as occurring within the same SCL. A similar approach was used in a comparative study to show that 52% of the interacting protein pairs in humans tend to have same SCL [8]. Lee *et al. *[9] extended the network-based approach by complementing the classification with a 'Divide and Conquer k-Nearest Neighbour' (DC-kNN) approach, with increased SCL predictive ability in yeast. Previous researchers have shown the importance of highly connected metabolites in the evolution of biochemical pathways which govern the flow of mass and energy in an organism [10,11]. To the best of our knowledge, the metabolite-linked network has only been used by Wagner and Fell [11] to report a positive correlation between the evolutionary age of metabolites and their degree of connectivity. Oron *et. al *[12] used constraint-based modelling on the metabolic network for predicting enzyme SCL, specifically considering the cross-membrane metabolite transporters (i.e. proteins). Thus, metabolic network information has not been implemented for predicting protein SCL, compared to data from PPI networks. As a first step towards developing such a prediction methodology, we have carried out large-scale statistical analysis of the SCL information contained in PPI and metabolite-linked networks.

**Figure 1:**
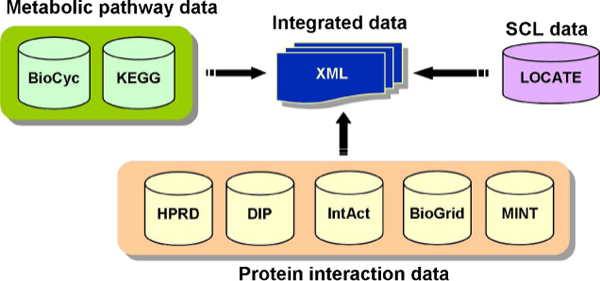
**Schematic representation of data integration**. Schematic representation of data integration. SCL information of LOCATE database integrated with that of interaction and metabolic data. The resulting integrated data is represented in XML format.

The availability of a large number of protein interaction and metabolic datasets from multiple databases has motivated us to conduct a statistical study to benchmark the predictive ability of localisation of human proteins, with respect to the various subcellular compartments. In this study, we collated PPI interaction and metabolite-linked protein interaction (metabolic information) from seven major databases and integrated these with the high quality SCL information present in the LOCATE database [13] (Figure 1; see Materials and Methods for details), to critically analyze the PPI and metabolic datasets for the SCL assignment of human proteins. Using experimentally validated physical interaction and metabolic datasets archived in various databases, we compared SCL annotations assigned by LOCATE with that of the Gene Ontology (GO) assignment for major subcellular compartments: cytoplasm (GO:0005737), cytoplasmic vesicle (GO:0016023), extracellular (GO:0005576), endoplasmic reticulum (GO:0005783), endosomes (GO:0005767), Golgi apparatus (GO:0005794), lysosomes (GO:0005764), mitochondria (GO:0005739), nucleus (GO:0005634), plasma membrane (GO:0005886) and tight junction (GO:0005923). Our results provide an estimate of the reliability of SCL predictive ability of human proteins in the absence of sequence and structural features using the high-throughput protein interaction and metabolic dataset.

## Results

**Figure 2:**
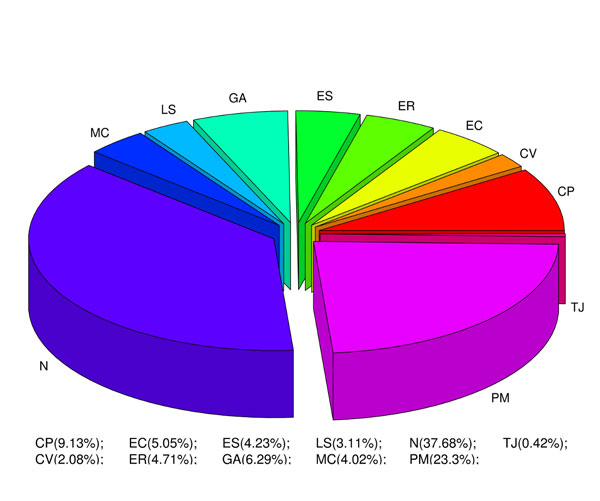
**Distribution of 6900 LOCATE proteins for various subcellular compartments**. The subcellular compartments are CP (cytoplasm), CV (cytoplasmic vesicle), EC (extracellular), ER (endoplasmic reticulum), ES (endosome), GA (Golgi apparatus), LS (lysosome), MC (mitochondria), N (nucleus), PM (plasma membrane), and TJ (tight junction).

As there is no specific database which combines protein interaction, metabolic and SCL information, we integrated data from independent individual databases containing pertinent information. The SCL data from LOCATE [13], PPI data from five interaction databases and metabolic data from two databases (Figure 1; details in materials and methods section) were integrated. LOCATE contains literature-curated SCL information for about 6900 human proteins (Figure 2) in various subcellular compartments. The distribution of proteins is not homogeneous across the various subcellular compartments, with proteins from some compartments such as the nucleus and the plasma membrane being over-represented. Therefore, we have carefully normalized the dataset, while measuring the statistical properties of our networks, to remove any bias toward specific SCL compartments.

Overall, 1,718 and 1036 proteins, respectively from the LOCATE dataset contain PPI and metabolic interactions. These reduced datasets were used for further analysis by considering the consistency of proteins across different databases and removal of the duplicate and redundant entries. For comparing the SCL assignment, we carefully merged low-level SCL annotation with that of the high-level SCL annotation mentioned in the GO hierarchy (see Additional file 1 for the merged GO-IDs). We used the same hierarchical level of SCL annotation for comparing LOCATE and GO annotations. Also, we will refer to the metabolite-linked protein interaction network as the metabolic network or MLPI, and the gene ontology annotation as GOA.

### Categorical analysis of protein pairs

In order to test, how protein pairs are localized within the same subcellular compartments, Pearson's χ^2 ^(chi-square) test was performed. This statistical test shows that χ^2 ^= 1270.19, *P-value *< 2.2 × 10^-16 ^for physically interacting protein pairs and χ^2 ^= 110.02, *P-value *< 2.2 x10^-16 ^for metabolite-linked protein pairs (Tables 2 and 3). Thus, the incorporation of PPI and metabolic data dramatically improve the significance of SCL prediction, while the confidence level in SCL predictions with PPI information is much higher than that with metabolic information. The contingency table for metabolic interaction revealed that the observed frequency of metabolite-linked protein pairs with the same SCL is 20.94% more compared to the expected value, whereas the same observation seem to be twice as much (93.35%) for physically interacting protein pairs. The number of interacting protein pairs having the same or different SCL is observed to be nearly the same as in the PPI network. However, the metabolic network has fewer metabolite-linked protein pairs with the same SCL compared to that with different SCL. From Tables 2 and 3, we have extracted 4136 physically interacting protein pairs from 1156 proteins and 4551 metabolically linked pairs from 509 proteins for network analysis.

**Table 2:** Chi-square test for physically interacting protein pairs.

	Pairs with same SCL	Pairs with different SCL	Row total
**Physical interaction present**	2081 (1076.26)	2055 (3059.74)	4136
**Physical interaction absent**	381716 (382720.74)	1089051 (1088046.26)	1470767
**Column total**	383797	1091106	1474903
**Chi-square (χ^2^) Value: 1270.192**	***P-Value*: < 2.2 × 10^-16^**		

A 2 × 2 contingency table, showing the distribution of direct physical interaction of protein-pairs, as the observed number of pairs and the expected values (assuming independence) shown in parenthesis.

**Table 3:** Chi-square test for the metabolite-linked protein pairs.

	Pairs with same SCL	Pairs with different SCL	Row total
**Metabolite-linked Pairs**	1465 (1158.12)	3086 (3392.88)	4551
**Non-metabolite-linked Pairs**	132345 (132651.88)	388929 (388622.12)	521274
**Column total**	133810	392015	525825
**Chi-square (χ2)- Value: 110.02**	***P-Value*: < 2.2 × 10-16**		

A 2 × 2 contingency table, showing the distribution of metabolite-linked protein pairs, as the observed number of pairs and the expected values (assuming independence) in parenthesis.

### Interaction between various subcellular compartments

**Figure 3:**
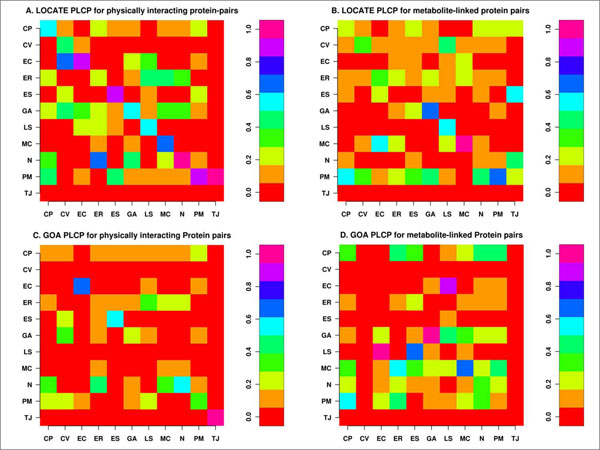
**Protein paired localisation correlation profile**. Paired Localisation Correlation Profile (PLCP) for LOCATE and GOA SCLs for major subcellular compartments for the physically interacting or metabolite-linked protein pairs. The subcellular compartments are CP (cytoplasm), CV (cytoplasmic vesicle), EC (extracellular), ER (endoplasmic reticulum), ES (endosome), GA (Golgi apparatus), LS (lysosome), MC (mitochondrion), N (nucleus), PM (plasma membrane), and TJ (tight junction). **A **and **B **are LOCATE SCL correlation profiles, whereas **C **and **D **are GOA correlation profiles.

We measured the statistical significance of SCL correlation profile based on the Paired-Localisation Conditional Probability (PLCP; see Methods section for details), for both the LOCATE (manually curated from the literature) data as well as the GOA assigned SCL (excluding electronic annotation, which is automatically-assigned evidence code). Figure 3 shows significant correlation along the diagonals suggesting that the interacting protein pairs tend to co-localize in the same compartment. Comparing the LOCATE-assigned SCL (Figure 3A), we observe a strong correlation for physically interacting protein pairs to occupy the same compartment in the cytoplasm (CP), cytoplasmic vesicles (CV), extracellular (EC), endosomes (ES), Golgi apparatus (GA), lysosome (LS), mitochondrion (MC), nucleus (N) and plasma membrane (PM). The same comparison on the GOA SCL (Figure 3C) shows conservation for EC, ES, GA, MC, N, PM and TJ. We also observed significantly strong correlation of nuclear proteins (Figures 3A and 3C) to interact with proteins found in cytoplasm, ER and Golgi for the LOCATE dataset and the cytoplasm, ER and mitochondrion for the GOA dataset. Similarly, plasma membrane proteins show significant interaction with the proteins in the several other subcellular compartments (Figures 3A and 3C).

The MLPI profile shows strong correlation of interacting protein pairs to have same SCL for GA, LS and MC. LOCATE data suggests significant correlation of metabolite-linked interaction of PM proteins with those in other compartments. Overall, the GOA dataset shows significant interaction across compartments in comparison to that of the LOCATE dataset (Figures 3B and 3D).

**Figure 4:**
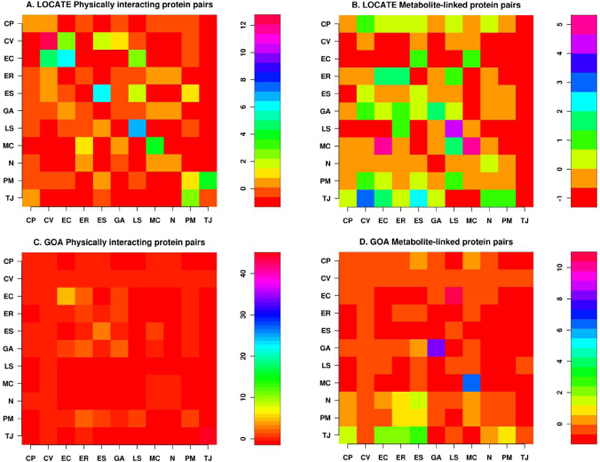
**Z-score correlation profile**. The Z-score correlation for LOCATE and GOA SCLs in the major subcellular compartments (see Additional file 1 for details) for the physically interacting and metabolite-linked protein pairs. A and B are LOCATE SCL correlation profiles, whereas C and D are GOA correlation profiles. Refer to Additional file 2 for Z-score values.

We further tested the hypothesis of whether the network of interacting protein pairs is different from a random network, by calculating the Z-score between the given compartments (described in the Methods section). The random network was simulated by rewiring the network such that the degree associated with each node in the real network remains the same [14]. The *P*-value can then be obtained by comparing the Z-score to a standard normal distribution. Comparing with a "properly" randomized network ensemble (1000 in our case) allows us to concentrate on those statistically significant localisation patterns of these complex interaction networks that are likely to reflect the conserved interaction pairs across different subcellular compartments. The statistical significance of correlation profiles were calculated for PPI and metabolic networks for each paired compartments. The Z-score profile scales differently for the physically interacting and metabolite-linked protein pairs (Figure 4). The PPI network Z-score (Figures 4A, C) suggest that compared to random networks, the number of interacting protein pairs co-locating in the same compartment is significant for EC (*P-value *< 9.8 e-10), MC (*P-value *< 3.7 e-05), LS (*P-value *< 4.5 e-12), ES (*P-value *< 1.8 e-09) and CV (*P-value *< 1.9 e-35) for the LOCATE dataset (Figure 4A and Additional file 2). We also observed a significant correlation for CV proteins to interact with EC proteins (*P-value *< 5.4 e-06) but not otherwise i.e. EC proteins do not interact with CV proteins at a significant *P-value *< 0.01. Similarly, TJ proteins are more likely to interact with that of the PM proteins (*P-value *< 4.3e-05), whereas the likelihood of PM proteins to interact with TJ proteins is less significant (*P-value *~ 0.01). GOA SCL assignment (Figures 4C) suggests that statistically significant protein pair interactions occur within TJ (*P-value *~ 0) and EC (*P-value *< 1.36e-07). Proteins pairs within the ES compartment seems to have a weak interaction (*P-value *~ 0.0007). Similar weak interactions have been noticed between the proteins in the ER compartment with those of the GA (*P-value *~ 0.007) (Additional File 2).

The metabolic Z-score correlation profile suggests a strong correlation of metabolite-linked protein pairs to have the same SCL within MC (*P-value *< 6.0e-07) and LS (*P-value *< 4.7e-05) in the LOCATE dataset (Figure 4B), while the GOA SCL (Figure 4D) assignment suggests the same for GA (*P-value *< 1.0e-15) and MC (*P-value *< 1.3e-10). A statistically significant proportion of EC proteins interacts with MC proteins (*P-value *< 1.0e-05) for the LOCATE SCL (Figure 4B). In the GOA dataset, LS proteins interact with EC proteins (*P-value *< 1.1e-26; Figures 4D). The detailed description of paired-compartment Z-scores and calculated *P-values *are available from Additional File 2.

### Analysis of PPI and Metabolic Networks

**Figure 5:**
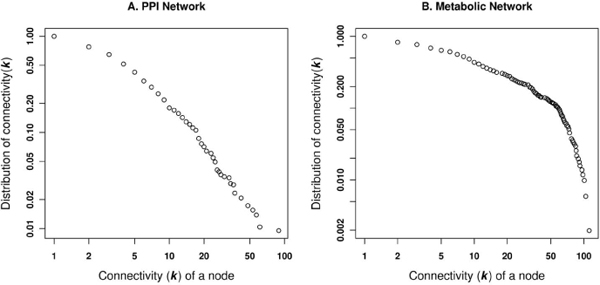
**Network connectivity or degree distribution**. The cumulative frequency distribution of the connectivity as a function of the connectivity or degree (k) is presented for A. PPI network and B. Metabolic network.

To track the variation in structural topology between PPI and metabolic networks, we analyzed their topological properties of both the networks for human proteins in integrated dataset (Figure 1). The interaction network used in this study consists of 4136 direct physical interactions between 1156 human proteins (Table 2), whereas the metabolic network consists of 4551 interactions between 509 proteins (Table 3). This suggests that the metabolic network is denser with more edges between the protein nodes. Both the protein interaction network and the MLPI network belong to the class of scale-free networks, suggesting that both networks evolved by adding new nodes to existing highly connected nodes. In these networks, the number of nodes with a given number of neighbours (connectivity, *K*), scales as *P(K) *α 1/*K*^γ^. The plot of the connectivity can be fitted by a power law, where γ = 1.52 and γ = 1.34, respectively for the physically interacting and metabolite-linked protein pairs (Figure 5A and 5B).

**Figure 6:**
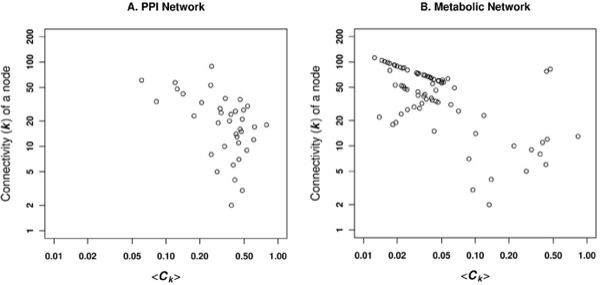
**Average clustering against the node degree**. The average clustering coefficient (* < C_k _>*) for each degree/connectivity, showing the probability that the adjacent neighbouring nodes of a node are connected is plotted as a function of the node degree in A. PPI network and B. Metabolic network.

The connectivity probability of nodes and its nearest neighbours are the same compared to the connectivity of any of the nodes chosen randomly, in a random network. On the other hand, a real network comprises an ordered lattice which is extended as the network grows, i.e. some order is achieved depending on how the co-ordinates of each new node are added, with respect to that node's neighbours (clusters) and independent of the total number of nodes present in the network [15]. Therefore, we have calculated the average clustering coefficient (*** < C_k _>***) associated with the given degree in PPI and metabolic networks, to study the global network topology. The PPI network shows random but gradual decrease of larger values of *** < C_k _>***associated with the high degree protein nodes. This simply means that the highly connected protein nodes are not connected, i.e. protein hubs are not connected, which is a specific signature for the non-modular nature of any real network (Figure 6A) [16]. The metabolic network, on the other hand, shows linear variation of highly connected nodes for the lower range of *** < C_k _>***associated with the higher degree nodes, implying the existence of hierarchical or modular structures (Figure 6B) [16,17].

**Figure 7:**
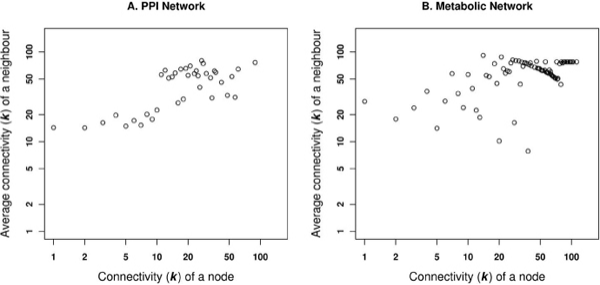
**Average connectivity of a neighbouring nodes**. Correlation in the connectivity of neighbours, with respect to a specific node of a given degree in A. PPI network and B. Metabolic network.

Assortativity measures the collaboration of similar entities to achieve a single goal, whereas a disassortative nature suggests the association of different entities to achieve the same goal. Therefore, to observe the assortative or disassortative nature of human PPI and metabolic networks, we calculated the average degree of the neighbouring proteins as a function of the each nodes degree [18]. For the PPI network, Figure 7A shows an increase in the neighbouring node degrees associated with higher degree nodes. This topological behaviour is the characteristic signature of the assortative network, thus suggesting that PPI is an assortative network. This observation is absent in the metabolic network (Figure 7B), where there is a decrease in the association with the high degree neighbours for the high degree nodes, i.e. nodes with the high degree *k *tend to be disconnected on an average, to others of lower degree. The power-law exponents (γ) for the degree assortativity are 1.2 and 1.1 in PPI and metabolic networks, respectively.

**Figure 8:**
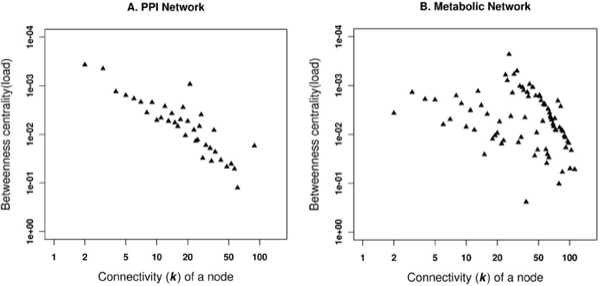
**Correlation between connectivity of nodes and betweenness centrality**. Plots showing the correlation of the betweenness centrality associated with the connectivity (*k*) of nodes for A. PPI network and B. Metabolic network.

We have also calculated the betweenness centrality, to measure the load in our PPI and metabolic networks [19]. This measurement is commonly used in sociology to quantify the influence of a person in a society. In our case, it helps to quantify the information carrying capacity of a specific protein in the network. The PPI network shows a linear behaviour of the centrality measure associated with the connectivity of a node (*k*), whereas the metabolic network has a non-linear, random behaviour (Figure 8).

Figures 6 and 7 together indicate that the metabolic networks can be characterized with high degree nodes interconnecting highly connected subgraphs, but with no or few connections among nodes in different subgraphs. This implies that the metabolic pathways are inter-connected via substrates between different compartments. Table 4 provides data on other topological features of the networks.

**Table 4:** Topological characteristics of PPI and metabolic networks.

	*Protein interaction network*	*Metabolic network*
*Number of nodes*	1156	509
*Number of edges*	4136	4551
*Clustering coefficient*	0.29	0.05
*Average clustering coefficient*	0.40	0.16
*Average path length *	4.77	4.09
*Diameter*	13	14

### Network-based neighbours for example proteins

From the normalized datasets that we have studied, of the many biologically relevant proteins, we have presented two specific examples. The first example is of a protein which specifically interacts with proteins co-located in the same SCL, while the second protein has interaction partners in different SCLs.

**Figure 9:**
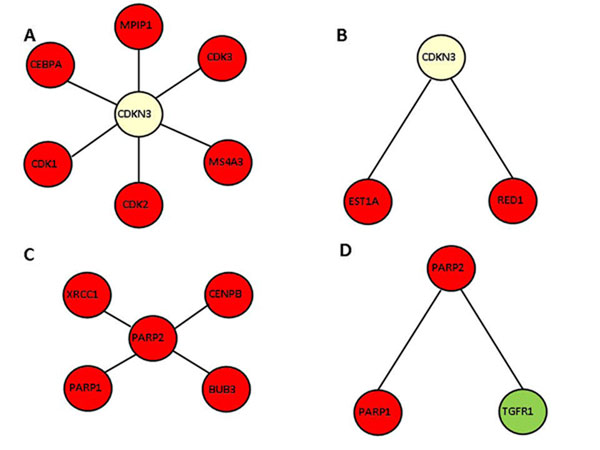
**Examples showing the neighbouring proteins of CDKN3 (located in the perinuclear region of cytoplasm) and PARP2 (nuclear protein) in the PPI and MLPI networks**. Proteins located in the nucleus, perinuclear region of the cytoplasm and plasma membrane are coloured in magenta, light yellow and light green respectively. Additional file 3 shows the differences in LOCATE and UniProt assigned SCL.

We examined the neighbouring proteins of human cyclin-dependent kinase inhibitor 3, CDKN3, in our PPI and MLPI networks (Figure 9). We note that this protein has been assigned the perinuclear region of the cytoplasm as SCL in UniProt, for a normal cell [20] (data available from Additional file 3). We found that CDKN3 is linked to double-stranded RNA-specific editase 1, RED1 and telomerase-binding protein, EST1A in our metabolic network, both interaction partners being located in the nucleus (Figure 9B). In the PPI network (Figure 9A), the same protein, CDKN3 is observed to interact with six proteins located in the nucleus: CDK2 (cell division protein kinase 2), MS4A3 (protein modulator of G1-phase to S-phase cell cycle transition), CDK3 (cell division protein kinase 3), MPIP1 (phosphatase protein inducer of mitotic progression), CEBPA (DNA-binding protein) and CDK1 (cell division protein kinase 1, required for the progression of S-phase and mitosis). As early as 1993, Gyuris *et al. *[21] have reported that CDKN3 is expressed at the G1-phase to S-phase transition during the cell division process and is known to form a stable complex with CDK2. Our network analysis clearly supports CDKN3 being located in the periplasmic space and interacting with neighbouring proteins in the nucleus due to the porous nature of the nuclear membrane (Figure 9A and 9B) and is consistent with our PLCP analysis results on the interaction, which show that the nuclear proteins seem to interact with proteins of the cytoplasm (Figure 3).

Subsequently, we examined the neighbouring proteins of human poly [ADP-ribose] polymerase 2 (PARP2) (Figure 9C and 9D). In the MLPI (Figure 9D), one of the interacting partners of PARP2 is TGF-beta receptor type-1 (TGFR1), which is a signalling molecule located in the plasma membrane. The other interacting neighbour is PARP1 (poly [ADP-ribose] polymerase 1) located inside the nucleus, which interaction alone is preserved in the PPI network (Figure 9C). Considering the integrated network approach of combining different networks, we can thus infer not only the SCL of the interacting proteins but also the biochemical signal *via *the plasma membrane, to identify the exact biological function of this polymerase, which is in accord with the earlier findings of Sharan and Ideker [22].

We have analyzed the SCL annotation of the 15 proteins in the above interacting pairs to determine the correlation of SCL assignment between LOCATE and UniProt databases (available in Additional file 3). We note that UniProt has no annotation for four proteins (27%), while two (13%) of the proteins have SCL assignments different from those in LOCATE. The remaining nine proteins have the same SCL assignments in both databases. These results support the use of experimentally determined SCL annotations from LOCATE for this analysis, over UniProt SCL assignments.

## Discussion

**Figure 10:**
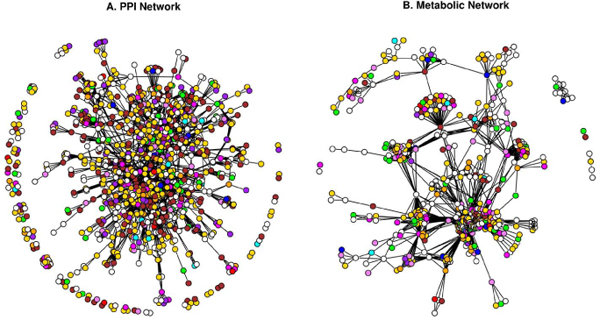
**Visualization of PPI and metabolic networks**. In the graphical representation of networks, the nodes and edges are represented by circles and lines, respectively. Circles representing the interacting proteins are coloured by the SCL compartment: cytoplasm (green), cytoplasmic vesicle (blue), endoplasmic reticulum (orange), endosome (red), extracellular (purple), Golgi apparatus (magenta), mitochondrion (violet), lysosome (cyan), nucleus (gold), plasma membrane (brown) and Tight junction (pink). White nodes represent proteins with unknown SCL and grey nodes represent proteins with multiple SCL.

Based on the topological comparison of networks, we were able to gain more insights into the structural differences in the PPI and metabolic networks of human proteins. Having shown that PPI and metabolic networks are scale-free, we further showed that the metabolic network is not assortative and modular (Figure 10).

The PPI network can be viewed as a network model where proteins collaborate on the number of cellular processes a single protein can handle at any time. This network model is evident from network behaviour with a power-law distribution *P(k) ~ k*^-γ ^where γ = 1.5 [23]. A similar observation is noted in the PPI network for passive interaction across subcellular compartments with γ = 1.52, due to the high false-positive rate. PPI data is known to have a high false-positive rate, i.e. the reliability of the possible observed interaction is questionable as with the high coverage rate. If a given protein interacts with a large number of other proteins, it is most likely a sticky protein and the observed interactions associated with this protein do not have a real functional association. Therefore, the passive interaction defines the unreliability of the observed interaction, which could happen by chance. The linear behaviour of betweenness centrality against the connectivity of node (*k*) in PPI network further suggests the presence of non-localized behaviour of interactions across compartments, compared to localized metabolite linkages among proteins inside the same subcellular compartments. This observation is also evident from the χ^2 ^statistics where the number of interacting protein pairs having the same localization is nearly the same as in different subcellular compartments (Table 2). We compared LOCATE assigned SCL with that of the GOA for the protein pairs across the different subcellular compartments, considering the multiple localisation for proteins. This comparison suggests significant differences among the annotation process (Figure 3A and 3C). The correlation profile (PLCP) suggests a strong correlation of interacting protein pairs within the same subcellular compartments. There is statistically significant cross-interaction among proteins in the nucleus with those of other cellular compartments. This is attributed to the fact that the nucleus has a porous cell membrane, which facilitates free diffusion and interaction between proteins across compartments. Subcellular compartments such as the Golgi apparatus, the endoplasmic reticulum and the lysosome indicate weak but significant correlation, which is in accord with the fact that the Golgi apparatus and the endoplasmic reticulum are inter-linked subcellular compartments for the translocation of proteins to various other compartments after the translation of mRNA to protein on the ribosome. The Z-score correlation profile for the PPI network shows that while interactions are conserved within compartments (along the diagonal, Figure 4A and 4C) with respect to the random network, there is also significant interaction of protein pairs across other subcellular compartments.

The metabolic network has an evolutionary constraint where only a few proteins are linked through common metabolites to maintain high substrate specificity in the higher eukaryotes [24]. Hence proteins are distributed in various subcellular compartments unlike prokaryotic proteins which contain co-evolving protein domains to carry out multiple tasks. Moreover, eukaryotic metabolic pathways are optimized via cross connections across subcellular compartments. This is revealed in the χ^2 ^statistics where few protein pairs have the same subcellular compartments compared with pairs from different compartments. PLCP suggest that protein pairs are not conserved for the compartments such as cytoplasm, cytoplasmic vesicles, endoplasmic reticulum and endosome (Figure 3B and 3D). This is due to the fact that the numbers of metabolite-linked protein-pairs are less and secondly, there are lots of dynamics happens among these compartments, as number of cellular pathway are distributed across compartments, hence it makes difficult to capture from our static picture of PLCP calculation. Even though the dynamics of some compartments are difficult to capture through the statistical measures, it is very useful to see how cellular processes are tightly controlled inside the subcellular systems such as mitochondrion and lysosome. The Z-score correlation profile of LOCATE and GOA SCL suggests that the metabolite-linked protein pairs seems to be more conserved across diagonals compare to that of randomized network and hence metabolite-linked interactions are tightly regulated within the same compartments (Figure 4B and 4D).

## Conclusions

The network analysis showed that there is significant difference between the topological properties measured in the human PPI and metabolic networks. Network comparison indicates the usefulness of metabolite-linked protein interaction (metabolic network) that can be used for the prediction of protein's SCL in the compartments such as mitochondria and lysosome. Our results lead to the observation that proteins in PPI network interact passively, whereas metabolic network evolve under evolutionary constrain to maintain substrate specificity. The series of analysis presented in this study suggests the applicability of metabolic (metabolite-linked protein interaction) network to explain the empirical data. The integrated network approach of using PPI and MLPI data developed here will provide a robust basis for predicting SCL for higher eukaryotes, along with the comparative network studies across species.

## Methods

### Data integration and construction of database

In the absence of a specialized database combining protein interaction, metabolic and SCL information, we have integrated data from independent individual databases. The LOCATE database contains SCL information from human and mouse proteins collected from both literature and direct experiment [13]. SCL data on human proteins from LOCATE database were integrated with the interaction data deposited in the PPI databases: HPRD [25], DIP [26], MINT [27], BioGRID [28] and IntAct [29]. Similarly, metabolic data (MD) were collected from the databases, KEGG [30] and HumanCyc [31] and integrated with the SCL data of the human proteins with the LOCATE database. This integrated dataset is recorded in XML format (Figure 1 and Additional file 4). LOCATE data contains 64,637 human proteins with known or predicted SCL information. Our integrated database contains 6,900 proteins with known SCL information curated from the literature (Figure 2). We used UniProt-ids and RefSeq-ids for consistent mapping across the three different datasets (i.e. SCL, PPI and MD).

### Identification and removal of inconsistency and redundancy

The LOCATE protein database [13] contains references to sequence databases such as UniProtKB [2] and RefSeq [32]. Protein entries with secondary accession were mapped to their primary identifiers mentioned in the protein sequence databases. RefSeq identifiers where used to extract UniProt identifiers where LOCATE entries contain RefSeq identifier but not the UniProt accession number. This allows consistent one-to-one mapping of protein entries across various databases. Duplicate entries of known protein interactions mentioned in PPI databases were carefully removed while analyzing interaction information in each LOCATE entry.

The metabolic linkage between proteins was established by considering only those compounds which occur in less than 50 reactions per compound in a given metabolic database. This ensures the removal of ubiquitous compounds such as ATP, NADH, H_2_O, H^+ ^etc. (see Additional files 5 and 6 for the lists of ubiquitous compounds). Ambiguous metabolites where removed, for example, HumanCyc reaction: GLUTATHION + **RX **< = > |S-Substituted-Glutathione| + **HX**, where RX and HX are ambiguous metabolites. Only those metabolites which contain unique compound-ids, were further considered for linking proteins, while those with generalized descriptions were omitted. E.g. General-Protein-Substrates and General-Phos-Protein-Substrates were not considered as linking metabolites shown in a reaction: |**General-Protein-Substrates**| + ATP < = > |**General-Phos-Protein-Substrates**|.

For the current study 1,718 and 1036 LOCATE proteins out of 6900 (literature curated), were linked *via *direct physical and metabolite-linked protein interactions, respectively. In the topological studies of PPI and metabolic networks, we considered 1156 and 509 proteins with 4136 and 4551 interactions respectively.

### Construction of networks

All LOCATE protein entries were linked *via *interactions (either physical or through a common metabolite) and the data were recorded in xml format (available from Additional file 4). This dataset was used to build the undirected networks using the R igraph package [33]. We used *degree *and *transitivity *functions for calculating the degree distribution and clustering coefficient in our networks. Random networks were generated by using the *rewire *function of the R igraph package.

### SCL analysis of the protein pairs

Correlation profiles were created using Paired-Localisation Conditional Probability (PLCP) for both PPI and metabolic networks [9]. This measure shows how the interacting protein pairs are distributed across various subcellular compartments. For a given protein in the compartment *C_i _*having an interacting partner in compartment *C_j_*, PLCP is defined as

(1) $$P\left(C_{i}|C_{j}\right)=\frac{C_{ij}}{\sum_{k}C_{jk}},$$

where *C_ij _*is the normalized number of interactions between protein pairs spanning compartments *C_i _*and *C_j_*. *C_ij _*is defined as:

(2) $$C_{ij}=\frac{\sum_{x\in C_{i},y\in C_{j}(x\neq y)}\frac{\lambda \left(x,y\right)}{N(x)+N(y)}}{N(C_{i})+N(C_{j})}$$

where, λ(*x*, *y*) is 1 if there is an interaction between proteins *x *and *y*, otherwise, 0. *N(Ci) *is the number of proteins in compartment *C_i _*and *N(x) *is the number of localisations known for protein *x*.

The Z-score correlation profiles were analyzed between interacting protein pairs from the real and random networks as given by:

(3) $$Z\left(Ci,Cj\right)=\frac{N{\left(Ci,Cj\right)}_{real}-\left\langle N{\left(Ci,Cj\right)}_{random}\right\rangle }{\sigma {\left(Ci,Cj\right)}_{random}}$$

where, *N*(*C_i_*, *C_j_*)*_real _*and ⟨*N*(*C_i_*, *C_j_*)*_random_*⟩ represent numbers of physically interacting or metabolite-linked protein pairs in real and random networks respectively. *σ*(*C_i_*, *C_j_*)*_random_*, represents the standard deviation in the ensemble of a 1000 random networks.

### Statistical validation of networks

We analyzed the topological property of PPI and metabolic network calculating the most significant network features, namely clustering coefficient, betweenness centrality, average path length, degree distribution and correlation profile calculation. For a graph *G *with *u *and *v *as two vertices, the path from *u *to *v *will pass sequentially through vertices *v*_1_, *v*_2_...*v*_k_, with *u *= *v*_1 _and *v *= *v*_*k*_, such that for *i *= 1,2.....*k*-1: (i) (*vi*, *vi*+1) ∈ *E*(*G*) i.e. the edges set and (ii) *vi *≠ *vj *for *i *≠ *j*. The path length is then said to be (*k*-1). The simple *geodesic distance*, *d*(*u*, *v*) from *u *to *v *is the length of the shortest path from *u *to *v *in the graph *G*. The average path length, ⟨*l*⟩, of such a graph is defined as the average of values taken over all the possible pairs of nodes connected by at least one path:

(4) $$\langle l\rangle =\frac{2}{N(N-1)}\sum_{u,v=1}^{N}l_{uv}$$

where, *N *is the number of nodes and *l_uv _*is the distance between two nodes, *u *and *v*. The diameter of the network is defined as the maximum distance between two nodes of a graph *G*, i.e. *D *= max{*d_uv _*|*u*, *v *∈ *N*}, where *N *is the total number of nodes in the graph or network.

The clustering coefficient is another characteristic of a network which is unrelated to the degree distribution. It is a quantitative measure to the proximity of the neighbourhood of each node to form a complete subgraph (clique) and thus defines a measure of the local behaviour of the small world network [34]. The clustering coefficient is defined as,

(5) $$Ci=\frac{2K}{ki(ki-1)}$$

where, *K *denotes the sum of the neighbouring pairs among the *k_i _*nodes connected to the node *i*. Similarly, one can define an average clustering coefficient as,

(6) $$\langle C\rangle =\frac{1}{K}\sum_{i=1}^{K}Ci$$

Centrality is one of the key structural aspects of the nodes in a network and is a measure of the relative influence of each node on the network. We calculated betweenness centrality, which is the fraction of shortest paths between all the pairs of nodes that passes through a given node [19].

## Competing interests

The authors declare that they have no competing interests.

## Authors' contributions

GK designed the experiment, analysed the data and wrote the first draft of the manuscript. SR directed this study and finalized the manuscript.

## Supplementary Material

Additional file 1 **Merged list of subcellular compartments for the LOCATE and GOA SCL**. This contains the list of compartment at the lower-level of GO hierarchy which were merged with that of the higher level of GO cellular compartments for the analysis of major subcellular compartments. Click here for file

Additional file 2 **List of Z-score values for the paired SCL**. This contains the Z-score values and their calculated P-values for the paired compartments in the PPI and metabolic dataset, as described in Figure 3. Click here for file

Additional file 3 **SCL assignment of example proteins in Figure **9. The LOCATE SCL information compared to SCL annotations from the UniProt database. For each protein, the description, HGNC gene name and UniProt identifier are also provided. Click here for file

Additional file 4 **Integrated data**. This contains the LOCATE proteins with SCL information integrated with that of the PPI and metabolic dataset, as described in Figure 1. Click here for file

Additional file 5 **List of KEGG compounds per reaction**. A list of compounds from the KEGG database [30] with the number of known reaction. Click here for file

Additional file 6 **List of HumanCyc compounds per reaction**. A list of compounds from the HumanCyc database [31] with the number of known reactions. Click here for file
