# Supplementary material for: Network analysis of human protein location
Source: BMC Bioinformatics. 2010 Oct 15;11(Suppl 7):S9. doi: 10.1186/1471-2105-11-S7-S9 (PMC2957692; doi:10.1186/1471-2105-11-S7-S9)
Supplement: Additional file 3 — SCL assignment of example proteins in Figure 9. The LOCATE SCL information compared to SCL annotations from the UniProt database. For each protein, the description, HGNC gene name and UniProt identifier are also provided. [file 1471-2105-11-S7-S9-S3.doc]

**Table S3: SCL assignment of proteins in Figure 9.**

**Network Analysis of Human Protein Location**

Gaurav Kumar and Shoba Ranganathan

| Protein  Description | Gene name  (based on  HGNC [1]) | UniProt  ID | *LOCATE*  *SCL* | *UniProt*  *SCL* |
| --- | --- | --- | --- | --- |
| Cyclin-dependent kinase  inhibitor 3 | CDKN3 | *Q16667* | Cytoplasm | Cytoplasm |
| Cell division protein kina*se 3* | CDK3 | *Q00526* | Nucleus | - |
| Membrane-spanning 4-domains  subfamily A member 3 | MS4A3 | *Q96HJ5* | Nucleus | perinuclear  region |
| Cell division protein kinase 2 | CDK2 | *P24941* | Nucleus | - |
| Cell division protein kinase 1 | CDK1 | *P06493* | Nucleus | Nucleus |
| CCAAT/enhancer-binding protein  α  | CEBPA | *P49715* | Nucleus | Nucleus |
| M-phase inducer phosphatase 1 | MPIP1 | *P30304* | Nucleus | - |
| Telomerase-binding | EST1A | *Q86US8* | Nucleus | Nucleus |
| Double-stranded  RNA-specific editase 1 | RED1 | *P78563* | Nucleus | - |
| Poly [ADP-ribose]  polymerase 2 | PARP2 | *Q9UGN5* | Nucleus | Nucleus |
| Major centromere  autoantigen B | CENPB | *P07199* | Nucleus | Nucleus |
| Mitotic checkpoint  protein BUB3 | BUB3 | *O43684* | Nucleus | Nucleus |
| Poly [ADP-ribose]  polymerase 1 | PARP1 | *P09874* | Nucleus | Nucleus |
| DNA repair protein  XRCC1 | XRCC1 | *P18887* | Nucleus | Nucleus |
| TGF-beta receptor type-1 | TGFR1 | *P36897* | Plasma  membrane | Membrane |

1. Bruford EA, Lush MJ, Wright MW, Sneddon TP, Povey S, Birney E**: The HGNC Database in 2008: a resource for the human geno**me*. Nucleic Acids Re*s 2008**,** 36(Database issue):D445-448.
